# Transglutaminase 2 in breast cancer metastasis and drug resistance

**DOI:** 10.3389/fcell.2024.1485258

**Published:** 2024-10-31

**Authors:** Mengxin Li, Xuanzhong Wang, Jinghui Hong, Juanjuan Mao, Jiasi Chen, Xuyang Chen, Ye Du, Dong Song

**Affiliations:** ^1^ Department of Breast Surgery, General Surgery Center, The First Hospital of Jilin University, Changchun, China; ^2^ Department of Radiation Oncology, The First Hospital of Jilin University, Changchun, China; ^3^ Key Laboratory of Pathobiology, Ministry of Education, Department of Pathophysiology, College of Basic Medical Sciences, Jilin University, Changchun, China; ^4^ School of Basic Medicine and Life Sciences, Hainan Medical University, Haikou, China

**Keywords:** transglutaminase 2, epithelial to mesenchymal transition, metastasis, drug resistance, breast cancer

## Abstract

Transglutaminase 2 (TG2) is a widely distributed multifunctional protein with various enzymatic and non-enzymatic activities. It is becoming increasingly evident that high levels of TG2 in tumors induce the occurrence of epithelial to mesenchymal transition (EMT) and the acquisition of stem cell-like phenotypes, promoting tumor metastasis and drug resistance. By regulating intracellular and extracellular signaling pathways, TG2 promotes breast cancer metastasis to lung, brain, liver and bone, as well as resistance to various chemotherapy drugs including docetaxel, doxorubicin, platinum and neratinib. More importantly, recent studies described the involvement of TG2 in PD-1/PD-L1 inhibitors resistance. An in-depth understanding of the role that TG2 plays in the progression of metastasis and drug resistance will offer new therapeutic targets for breast cancer treatment. This review covers the extensive and rapidly growing field of the role of TG2 in breast cancer. Based on the role of TG2 in EMT, we summarize TG2-related signaling pathways in breast cancer metastasis and drug resistance and discuss TG2 as a therapeutic target.

## 1 Introduction

Breast cancer is the most commonly diagnosed cancer in women and the leading cause of cancer death among women, according to the estimates from International Agency for Research on Cancer (Sung et al., 2021). The main treatment methods include surgery, chemoradiotherapy, endocrine therapy and immunotherapy. However, the prognosis of patients is still limited by metastasis and drug resistance, especially in patients with triple-negative breast cancers (TNBC). Tumor metastasis is a complex process involving the invasion of primary tumor cells into surrounding tissues, followed by their circulation through the bloodstream to distant organs where they can establish growth (Chaffer and Weinberg, 2011). In breast cancer, primary tumor cells can metastasize to distant organs such as the brain, lungs, and bones, resulting in poor survival outcomes and ultimately contributing to patient mortality. Epithelial to mesenchymal transition (EMT) is a complex dynamic process that enables cells to gain migratory and invasive properties, influencing embryonic development, wound healing, and cancer metastasis (Chaffer and Weinberg, 2011). During EMT, breast epithelial cells lose adhesion and acquire mesenchymal traits, which play a crucial role in tumor metastasis. In the meantime, breast cancer cells undergoing EMT display a number of characteristics associated with cancer stem cell phenotypes, which make them tolerant to chemotherapy (Lu and Kang, 2019). Thus, therapeutics strategies that targeting EMT could not only prevent tumor metastasis, but also increase the sensitivity of chemotherapeutics.

Transglutaminase 2 (TG2), also known as guanosine triphosphate (GTP) binding protein alpha-h (Gαh) or tissue transglutaminase (abbreviated as TGM2, tTG, or TGase 2), is the most complex and ubiquitous member of the transglutaminase enzyme family (Eckert et al., 2014). This family includes nine members including TG1, TG2, TG3, TG4, TG5, TG6, TG7, FXIIIa and Band4.2, each with unique functions and tissue distributions (Eckert et al., 2014). TG2 is a multifunctional enzyme widely expressed in various tissues, playing critical roles in biological processes such as apoptosis, extracellular matrix formation, transamidation deamidation, and cell signaling. Its activities include both enzymatic and non-enzymatic functions, such as protein cross-linking, G-protein activity, cell adhesion and migration. Since its discovery in 1957, TG2 has been implicated in a range of diseases across different systems due to its diverse activities. Since the discovery of TG2 in 1957, various enzymatic and non-enzymatic activities of TG2 have been described, accounting for its involvement in diseases of various systems. TG2-mediated tumorigenicity was reported in gastric cancer (Lei et al., 2018), lung cancer (Lee et al., 2018), colorectal cancer (Miyoshi et al., 2010), ovarian cancer (Shao et al., 2009), pancreatic cancer (Lee et al., 2016), liver cancer (Yamaguchi et al., 2017), breast cancer (Agnihotri et al., 2013), leukemia (Csomós et al., 2010), prostate cancer (Han et al., 2014), renal cancer (Erdem et al., 2015), glioblastoma (Gundemir et al., 2017), meningiomas (Yuan et al., 2008), cervical cancer (Gupta et al., 2010), epidermal squamous cell carcinoma (Fisher et al., 2015), oral squamous cell carcinoma (Lim et al., 2017), mesothelioma (Zonca et al., 2017), melanoma (Di Giacomo et al., 2009), esophageal cancer (Leicht et al., 2014), osteosarcoma (Li et al., 2018), laryngeal cancer (Jin et al., 2012). In the field of breast cancer, high TG2 level contributes to EMT, chemoresistance, metastasis as well as the acquisition of stem cell-like phenotypes (Zaltron et al., 2024). Recent reports also described the involvement of TG2 in PD-1/PD-L1 inhibitors resistance (Choi et al., 2020). An insight into the role that TG2 plays in EMT, metastasis and drug resistance can provide new targets for breast cancer treatments. In the next sections, the TG2-related signaling pathways in the metastasis and drug resistance of breast cancer will be summarized in detail.

## 2 TG2: structure and function

**FIGURE 1:**
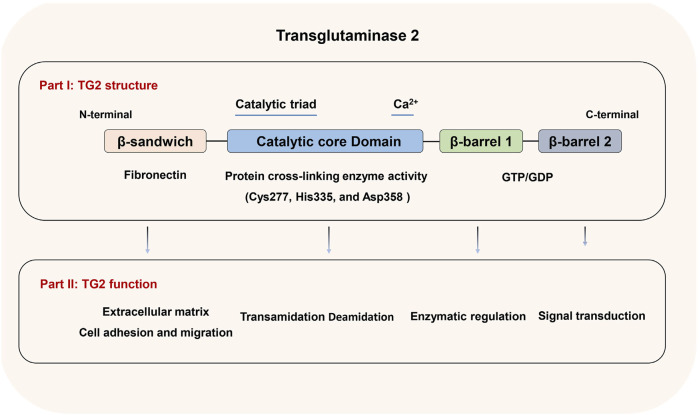
TG2 structure and function. A diagram of TG2 illustrating the β-sandwich, catalytic core, β-barrel1, and β-barrel2 domains, along with the biological roles linked to each domain.

Structurally, TG2 is composed of four key domains: the N-terminal β-sandwich domain, which has sites for fibronectin and integrin binding; a central catalytic domain that houses the catalytic triad (Cys277, His335, and Asp358) responsible for its transamidase function; and two β-barrel domains, which contain sequences for GTP/GDP and phospholipase C binding (Mehta et al., 2010) (Figure 1). The structure of TG2 enables it to function not only as a Ca^2+^-dependent transamidase but also as a GTPase, deamidase, protein kinase, protein disulfide isomerase, and scaffold protein (Nurminskaya and Belkin, 2012). TG2 has a wide tissue distribution including cytosol, mitochondria, nucleus, cell surface, extracellular matrix and extracellular vesicles. TG2’s functions as a GTPase and transamidase are mutually exclusive due to its two conformations, which are primarily regulated by Ca^2+^ and GTP/GDP levels. When Ca^2+^ levels are low, TG2 adopts a closed or folded conformation, binds GTP/GDP, and participates in intracellular signaling. Conversely, at higher Ca^2+^ levels, TG2 transitions into an open or extended conformation, binds to Ca^2+^, and triggers its transamidase activity for protein cross-linking (Eckert et al., 2015). Moreover, the oxidation of TG2 can render its transamidase activity inactive in the open/extended conformation by promoting the formation of a stable disulfide bond between Cys370 and Cys371. This inactivation can be reversed by the protein cofactor thioredoxin (Agnihotri et al., 2013; Eckert et al., 2015). Given the extensive distribution and various enzymatic and nonenzymatic activities, TG2 plays function in numerous cellular processes including cell adhesion, migration, growth, proliferation, differentiation, ECM organization and turnover, exocytosis, apoptosis and autophagy. Besides its link to inflammatory disease, celiac disease, neurodegenerative disease, diabetes, tissue fibrosis, etc. TG2 is involved in the initiation, progression, metastasis and drug resistance of various cancers. The role of TG2 in different tumors and tumor environment has been elucidated in several reviews (Eckert et al., 2015; Eckert, 2019; Tempest et al., 2021). In the following sections, we summarized the specific mechanism of TG2 in regulating EMT and EMT-based metastasis and drug resistance of breast cancer.

## 3 TG2 distribution in breast cancer

The wide distribution of TG2 in tumor tissues determines its functional diversity. The expression of TG2 in human breast tumor tissue was first described by Hettasch JM team in 1996 (Hettasch et al., 1996). In normal mammary tissues, TG2 mainly locates in the endothelium and the ECM surrounding the ducts and maintains a low expression level. In breast cancer *in situ*, a marked increased expression of TG2 was detected in the ECM as well as the boundary between the tumor cells and the normal mammary tissue. Later studies successively reported the increased expression of TG2 in the stromal cells surrounding breast cancer *in situ* and deduced that increased expression of TG2 in stroma represented the host’s attempt to restrict tumor growth and prevent it from spreading to distant sites (Mangala et al., 2005; Bravaccini et al., 2014). In invasive breast carcinoma, higher TG2 expression was reported in both malignant breast epithelium and surrounding stroma, compared to normal tissue (Singer et al., 2006). Besides, the normal tissues adjoin to invasive ductal carcinoma displayed a 10-fold higher TG2 activity compared to the normal tissues adjoin to fibroadenoma and atypical ductal hyperplasia (Balestrieri et al., 2012). Existing data suggested that TG2 could act as an independent prognostic factor for clinical stage and overall survival of breast cancer patients (Xu et al., 2022). Nonetheless, the prognosis value of TG2 still requires further confirmation based on more samples. In conclusion, TGM2 not only represents a promising therapeutic target but also serves as a potential prognostic marker for breast cancer.

The expression of TG2 in metastatic breast cancer seems inconsistent in different organs. The metastatic lymph node tumors from patients with breast cancer showed significant higher levels of TG2 expression compared with the primary tumors from the same patients (Mehta et al., 2004). Shinde et al. reported that TG2 was upregulated in a breast cancer bone metastasis model (HME2-BM), compared with the parental HME2 cells (Shinde et al., 2022). Similar results were also reported in mouse models of lung metastatic breast cancer that TG2 expression levels were higher in metastatic lung tumor tissues compared to primary tumors (Shinde et al., 2020). However, the comparison of brain metastatic breast cancer cell line MDA-MB-231-BR and metastatic breast cancer cell line MDA-MB-231 without organ selectivity revealed that the mRNA and protein levels of TG2 were both downregulated dramatically in MDA-MB-231-BR compared with MDA-MB-231 (Dun et al., 2015). It was speculated that low level of TG2 contributed to destabilize the brain microenvironment and facilitated metastatic colonization, which needed further investigation.

TG2 is also localized to extracellular vesicle (EV), which has been increasingly recognized as a key player in tumor organotropic metastasis. Schwager et al. demonstrated that weakly migratory MDA-MB-231 cells could release TG2-rich cancer-derived microvesicles to activate fibroblasts and enhance cancer cell dissemination (Schwager et al., 2022). Shinde et al. also described a robust increase in the amount of TG2 and crosslinked fibronectin present within fractions derived from metastatic breast cancer cells. Treatment of tumor-derived EV containing TG2 promoted breast cancer cells growth in a 3D model mimicking pulmonary metastasis of breast cancer (Shinde et al., 2020). However, the level of TG2 in EVs and its correlation to breast cancer metastasis should be verified in more models.

## 4 TG2 functions in breast cancer progression

### 4.1 1TG2 mediates EMT in breast cancer

As the first step of tumor metastasis, EMT confers invasiveness, drug resistance, and tumorigenic phenotype. Under the orchestrated of various transcription factors such as Snails, Twist and Zeb, the polarized epithelial cells undergo multiple biochemical changes that enable them to acquire a mesenchymal phenotype. As a result, the epithelial markers (E-cadherin, occludin, cytokeratin, etc.) are usually downregulated, while the mesenchymal markers (N-cadherin, fibronectin, vimentin, etc.) are upregulated. Importantly, cancer cells that undergo EMT also display the phenotype of cancer stem cells (CSCs), which are believed to be involved in tumor onset, relapse, recurrence, and resistance to chemotherapy or radiotherapy due to their stem cell-like characteristics. Breast cancer stem cells are characterized by a CD44^+^/CD24^−^ antigenic phenotype and also display enhanced expression of EMT markers (Mani et al., 2008). The close association between EMT and breast cancer stem cells have been illustrated in previous reviews (Agnihotri et al., 2013; Eckert et al., 2015). The occurrence of EMT in breast cancer is usually accompanied with the acquisition of cancer stem cell traits and both of them correlate positively with the development of metastasis and chemoresistance.

TG2 mediates both EMT and cancer stem cells traits in mammary epithelial cells. By regulating Snail1, Zeb1, Zeb2, and Twist1 levels, TG2 induced transcriptional repression of E-cadherin and transactivation of fibronectin, N-cadherin and vimentin, which can be reversed by knockdown of TG2 with siRNA (Kumar et al., 2010). At the same time, TG2-induced EMT also confer stem cell-like, invasiveness, drug resistance and tumorigenic phenotype of breast cancer (Agnihotri et al., 2013). A recent study also demonstrated that TG2 induced EMT-based osseous metastasis by increasing expression of Zeb1 via miR-205 inhibition in mice (Seo et al., 2019). These evidences indicated that high expression of TG2 induces EMT and stem cell-like traits and thus contribute to the development of drug resistance and metastasis of breast cancer cells.

### 4.2 TG2 in breast cancer drug resistance

Intrinsic and acquired drug resistance limit the treatment effect of chemotherapy, targeted therapy and immunotherapy, which are important components of breast cancer systemic treatment. Although targeted therapy plays a crucial role in the treatment of breast cancer, research on TG2 in the context of targeted therapy is currently limited, and thus it will not be discussed separately. For a long time, reports of TG2 on drug resistance of breast cancer mainly focus on its involvement in doxorubicin (DOX) resistance. Recently, the role of TG2 in PD-1/PD-L1 resistance in breast cancer has been described, which makes TG2 a more intriguing topic for further studies.

#### 4.2.1 Chemotherapy resistance

The role of TG2 in breast cancer chemotherapy resistance can be summarized in two aspects: Endogenous TG2 levels within the tumor cells, which determine their inherent sensitivity to chemotherapeutic agents. The exogenous influence of chemotherapeutic treatment, which induces acquired chemoresistance by promoting TG2-mediated EMT within the tumor cells.

The intracellular level of TG2 correlates with the intrinsic chemoresistance in breast cancer cells. TG2 levels in DOX-resistant MCF7 (MCF-7/DOX) and MDA-MB-231 were significantly higher than those in DOX-sensitive MCF-7 and MDA-MB-468 cell lines (Kim et al., 2006). High level of TG2 also contributed to acquisition of neratinib resistance in metastatic HER2^+^ breast cancer cells (Shinde et al., 2022). Conversely, TG2-deficient MDA-MB-231 cells displayed higher sensitivity to DOX as well as docetaxel compared to TG2-sufficient MDA-MB-231 cells (Mehta et al., 2004). TG2 also plays function in multidrug resistance (MDR) of breast cancer. Silencing TG2 by siRNA transfection downregulated the expression of P-glycoprotein (P-gp), multidrug resistance protein (MRP) and lung drug resistance protein (LRP) at mRNA and protein levels in MCF7/DOX cells, which were accompanied with decreased stemness and enhanced DOX sensitivity (Cheng et al., 2020). The expression level of TG2 is probably determined by epigenetic modification of TG2 gene. It is reported that TG2 gene is often aberrantly hypermethylated in primary breast tumors, which accounts for its reduced expression. Incubation of MCF-7 cells with the DNA demethylating agent 5-aza-2′-deoxycytidine resulted in a robust increase in TG2 expression (Ai et al., 2008). A comparative study also revealed that the TG2 gene was hypomethylated in MCF-7/DOX and cisplatin-resistant MCF-7 cells compared to that in MCF cells (Chekhun et al., 2007).

TG2-mediated EMT also played a role in the acquired chemoresistance of breast cancer cells. Despite inhibiting the proliferation of tumor cells, DOX could also promote wound healing and invasion ability of breast cancer cells by inducing EMT via regulation several factors including TGF-β, NF-kB, Twist1, Snail (Chen et al., 2013; Li Q. Q. et al., 2011; Li et al., 2009; Li W. et al., 2011). Treatment with DOX resulted in the increase of TG2 levels in MCF-7 cells, the elevated TG2 interacted with vimentin to promote EMT and cell movement, which was abrogated by TG2 inhibitor NC9 (Bianchi et al., 2021).

#### 4.2.2 PD-L1 inhibitor resistance

Anti-PD-1/PD-L1 immunotherapy has shown good efficacy in various types of cancer. The binding of programmed death-protein 1 (PD-1) on the T cell surface and its co-inhibitory ligand (CCL2) on the tumor surface results in T cell exhaustion and promotes tumor survival. Blockade of PD-1 and PD-L1 can maximize cytotoxic T cell activity and eliminate tumor cells. Atezolizumab, a type of PD-L1 inhibitor, has been approved by the FDA for the combined treatment of triple negative breast cancer (Schmid et al., 2018; Adams et al., 2020). However, the PD-1/PD-L1 inhibitors resistance was discovered and limited the treatment effect. Existing reports indicated that TG2 contributed to PD-1/PD-L1 inhibitors resistances in two aspects. On one hand, High TG2 levels induced CCL2 and PD-L1 expression in TNBC cells via NF-κB/Akt pathways (Choi et al., 2020). As an inhibitory chemokine, CCL2 blocked T cell tracking into the tumor by binding to CCR2 on T cell surface. The TG2-induced CCL2 activation can still lead to weakened T cells activity and drug resistance even if PD-L1 was blocked by PD-1/PD-L1 inhibitors. It was proposed that TG2 could be a predictive marker to PD-L1 inhibitor-resistant TNBC patients (Choi et al., 2020). On the other hand, a TG2–dependent covalent CXCL12–keratin-19 (KRT19) heterodimer was described in breast cancer cells. The CXCL12–KRT19 complex coat tumor cells, restrict T cell motility and result in PD-1/PD-L1 inhibitors resistances. Mechanistically, intracellular TG2 bound to kartin-19, a type of intermediate filament protein. The TG2-KRT19 complex was secreted to extracellular matrix, where it interacted with CXCL12 and formed filamentous CXCL12–keratin-19 covalent heterodimers (Wang et al., 2022). These discovers provide a good prospect for the role of TG2 in PD-1/PD-L1 inhibitor resistance, which requires further verifications in different models.

### 4.3 TG2 and metabolism

Metabolic reprogramming is an important mechanism for tumor cells to adapt to their microenvironment and promote their own survival and proliferation. Warburg effect, which is the preference for aerobic glycolysis even in the presence of oxygen, is the most significant manifestation of metabolic reprogramming in tumor cells. Existing studies showed that breast cancer cells with high TG2 expression exhibited enhanced glycolysis level, which was characterized by decreased oxygen consumption rates and increased extracellular acidification rates under normoxic condition (Lai et al., 2017). Mechanistically, TG2 plays a significant role in promoting glucose metabolic processes by catalyzing post-translational modifications of crucial enzymes, although it does not participate in cellular metabolic functions directly. A range of metabolic enzymes that are integral to glycolysis and the tricarboxylic acid cycle, including fructose-1,6-diphosphate aldolase, acid citrate hydrolase, lactate dehydrogenase (LDH), and glycerol-3-phosphate dehydrogenase (G3PDH), have been recognized as substrates for TG2 (Lai et al., 2017). Meanwhile, activation of the TG2/NFκB pathway enhances HIF-1α expression, leading to increased glucose uptake and lactate production, while simultaneously reducing mitochondrial oxygen consumption (Kumar et al., 2014). Recent studies have also shown that the TG2 enhanced glycolysis by regulating the MEK/ERK/LDH pathway in breast cancer cells (Xu et al., 2022). These studies suggest that TG2 plays an important role in promoting glycolysis in breast cancer. Further research is warranted to elucidate the precise mechanisms by which TG2 regulates glycolysis and to explore the potential therapeutic applications of TG2 inhibitors in countering metabolic reprogramming in breast cancer cells.

### 4.4 Role of TG2 in apoptosis

Most chemotherapy drugs exert their anti-tumor effects by inducing apoptosis in tumor cells. However, some patients experience poor prognoses due to anti-apoptotic mechanisms, with EMT and glycolysis being classic pathways for apoptosis resistance. As mentioned above, TG2 is overexpressed in drug-resistant and metastatic cancer cells, it confers apoptosis resistance by promoting EMT and glycolysis. Meanwhile, as a downstream target of HIF1α, TG2 has also shown to inhibit apoptosis by modifying the activities of key apoptotic proteins, such as caspase-3 and Bax (Cho et al., 2010; Jang et al., 2010; Yoo et al., 2012). However, TG2 was found to promote apoptotic process in several studies. It mediated the apoptosis triggered by calcium ionophore A23187 in cancer cells (Fok and Mehta, 2007; Oliverio et al., 1999). It seems that the intracellular localization and specific isoforms of TG2 are critical in determining its influence on cell fate (Antonyak et al., 2006; Milakovic et al., 2004). A recent study utilized TG2 inhibitors with different membrane permeabilities to analyze transcriptome changes in aggressive triple-negative cell lines (Ancona et al., 2024). The study revealed that the membrane-permeable inhibitor AA9 induced apoptosis by modulating signaling pathways related to EMT and glycolysis (Gallo et al., 2023). In contrast, the impermeable inhibitor NCEG2 stimulated the expression of ATP synthase and DNA replication proteins, potentially promoting cell proliferation.

These findings suggest that TG2 plays a complex role in the regulation of apoptosis, exhibiting both anti-apoptotic and pro-apoptotic effects that depend on the cellular context and its localization. The development of drugs capable of targeting intracellular TG2 may help overcome the apoptotic resistance observed in tumors.

### 4.5 Role of TG2 in autophagy

There is no direct evidence that proves the involvement of TG2 in breast cancer cell autophagy. However, several key autophagy-related factors showed interesting correlation with TG2. Previous reports demonstrated that TG2 can interact with phospholipase-Cdelta1 (PLCD1) via its GTP-binding activity and the TG2/PLCD1 complex regulated the invasive ability in TNBC cells (Huang et al., 2017). Recently, it is reported that TG2 levels was positively correlated with the phosphorylation of Akt and mTORC1 but negatively correlated with autophagy. The TG2/PLCD1 interaction promoted autophagosome degradation by activating the Akt/mTORC1 pathway (Lin et al., 2020). Another report indicated that p70S6K, a downstream target of the PI3K/Akt/mTOR pathway, played an important role in metastasis by regulating various key factors including TG2. Targeting p70S6K by shRNA inhibited orthotopic tumor growth and lung metastasis in animal model, which was accompanied with reduced TG2 level (Akar et al., 2010). These findings preliminarily suggested that high levels of TG2 correlate with low autophagy levels and inducing autophagy in breast cancer cells might be beneficial to prevent tumor metastasis.

## 5 TG2-regulated signaling pathways in metastasis and drug resistance

**FIGURE 2:**
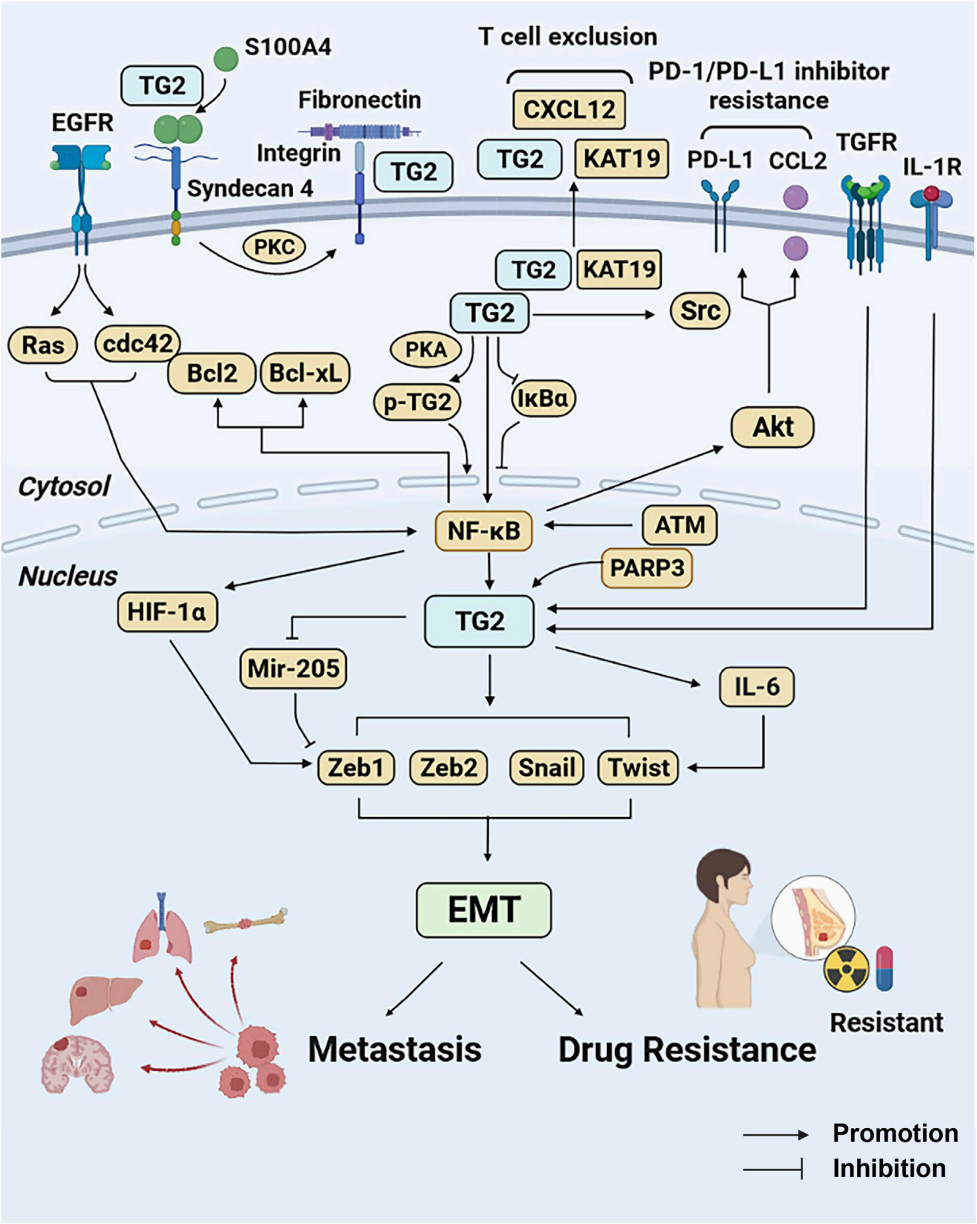
TG2-related signaling pathways in breast cancer metastasis and drug resistance. TG2 mediates NF-κB activation by causing IκBα degradation or being phosphorylated by PKA. In contrast, NF-κB promotes TG2 activation under the regulation of ATM. The NF-κB activation induced by TG2 promotes HIF-1α-mediated EMT and confers drug resistance by regulating Bcl-2 and Bcl-xL activities. Under the regulation of PARP3, TNFR, IL-1R and EGFR pathways, TG2 confers EMT by regulating EMT-related genes (Snail1, Zeb1, Zeb2, and Twist1) directly or bypass IL-6 and mir-205. TG2 also activates Src tyrosine kinase through its interaction with keratin-19, resulting in aberrant cell growth and enhanced tumorigenicity. The TG2-KRT19 complex secreted to extracellular matrix interacts with CXCL12 and forms filamentous CXCL12–keratin-19 covalent heterodimers and induces T cell exclusion. High TG2 expression induces PD-1/PD-L1 resistance by promoting CCL2 and PD-L1 via NF-κB/Akt pathways. In addition, extracellular S100A4 can be cross-linked directly by TG2. The syndecan-4 binds to S100A4 polymers and activates TG2 via PKC. The integrin-associated TG2 interacts with fibronectin and promotes fibronectin-mediated invasion. Abbreviations: TG2, transglutaminase 2; NF-κB, nuclear transcription factor-κB; PKA, protein kinase A; ATM, ataxia telangiectasia mutated kinase; HIF-1α, hypoxia inducible factor-1; EMT, epithelial to mesenchymal transition; Bcl-2, B-cell lymphoma-2; Bcl-xL, B-cell lymphoma-xL, PARP3, poly (ADP-ribose) polymerase 3; TGFR, Transforming growth factor receptor; IL-1R, interleukin 1 receptor; EGFR, epidermal growth factor receptor; KRT-19, keratin-19; PD-1, programmed death-protein 1; PD-L1, programmed death-protein ligand 1; CCL2, CC-motif chemokine ligand 2; PKC, protein kinase C.

TG2 promotes EMT-based metastasis and drug resistance of breast cancer via various extracellular and intracellular pathways. The schematic diagram of TG2-regulted signaling pathways has been summarized in Figure 2.

### 5.1 Extracellular TG2

Extracellular TG2 was reported to form various complexes by interacting with several kinds of transmembrane proteins and ECM component (Nurminskaya and Belkin, 2012). Only a few proteins have been confirmed in breast cancer cells. It was reported the TG2 on the surface of MDA-MD-231 cells was closely associated with integrins β1, β4 and β5, and contributes to fibronectin (Fn)-mediated cell attachment, survival and invasion (Mangala et al., 2007). As a member of the Ca^2+^-binding protein S100 family, S100A4 can act as either an intracellular or extracellular protein to accelerate cell migration. It was confirmed that S100A4 was a substrate for TG2. Extracellular S100A4 can be crosslinked directly by TG2 and the S100A4 polymers then activate the syndecan-4/PKCα/α5β1 integrin signaling pathways (Wang and Griffin, 2013), which promoted tumor cell migration and metastasis. The crosslinking of S1004A as well as S100A4-enhanced cell migration can be abolished by TG2 specific inhibitor R294. Recently, TG2 has been reported to participate in the formation of filamentous CXCL12–keratin-19 covalent heterodimers that coat tumor cells and mediate T cell exclusion in breast cancer tissues, which has been mentioned above in 5.2. The interactions of TG2 with platelet-derived growth factor receptor (PDGFR), vascular endothelial growth factor (VEGF), low density lipoprotein receptor (LDLR), G protein-coupled receptor 56 (GPR56), matrix metalloproteinase2 (MMP2), milk fat globulin EGF factor 8 (MFG-E8) were identified in various cancer cells, but have not been reported in breast cancer field, which provide the reference for further study.

### 5.2 Intracellular TG2

To illustrate TG2-related signaling clearly, we summarized intracellular TG2-related signaling cascades into four main pathways including NF-κB, EGFR, TGF-β and autophagy. It is worth mentioning that these pathways are not mutually exclusive. In contrast, they interact with each other and contribute to the EMT-based metastasis and drug resistance.

#### 5.2.1 Role of TG2 in NF-κB pathway

Existing studies have described the molecular feedback loop of TG2 and NF-κB clearly (Kim et al., 2006; Kumar and Mehta, 2012; Park et al., 2009; Kim et al., 2010; Ai et al., 2012). TG2 could activate NF-κB via two main pathways. On one hand, cytoplasmic TG2 can bind to IκBα and result in its degradation via non-proteasomal pathway. On the other hand, phosphorylation of TG2 at Ser216 induced by PKA also contributes to TG2-mediated NF-κB activation (Wang et al., 2012). On the contrary, under the stimuli of DNA damage response or oxidative stress, NF-κB is activated by ataxia telangiectasia mutated (ATM) and the NF-κB subunits p65 triggers TG2 transcriptional activation by interacting with TG2 promoter (Alpay et al., 2015). In fact, the baseline expression of TG2 is under the transcription regulation of NF-κB in cultured breast cancer cells, because pharmacological inhibition or knockdown of NF-κB resulted in a significant diminishment in TG2 expression in MCF-7/DOX and MDA-MB-231 cells (Ai et al., 2012).

The NF-κB activation induced by TG2 promotes breast cancer EMT and drug resistance via several pathways. The activated p65/p50 subunits could combine with TG2 and translocate to the nucleus, where the p65/p50/TG2 complexes bind to HIF-1α promoter and result in its increased expression. As a negative prognostic factor for breast cancer patients, HIF can not only induce EMT and promote metastasis as well as drug resistance by regulating Zeb1, Zeb2, Snail and Twist (Kumar and Mehta, 2012), but also lead to increased glucose uptake, increased lactate production and decreased oxygen consumption by mitochondria (Kumar et al., 2014). Similarly, TG2 was also reported to upregulate glycolysis via regulation of MEK/ERK/LDH pathway in breast cancer cells (Xu et al., 2022). TG2-mediated NF-κB activation can also confer drug resistance by upregulating Bcl-2 and Bcl-xL activity in MDA-MB-231 cells (Kim and Park, 2009), or activating Akt pathway in MCF-7 and T-47D cells (Wang et al., 2012).

#### 5.2.2 Role of TG2 in EGFR pathway

EGFR overexpression alters signaling pathways that have an impact on proliferation, metastasis, invasiveness and chemoresistance in breast cancer (Shetty et al., 2021). TG2 is also a downstream mediator of EGF/EGFR pathway. Treatment of EGF in SKBR3 and BT-20 cells led to enhanced TG2 expression and activity, which was required for EGF-mediated doxorubicin resistance (Antonyak et al., 2004). Mechanistically, EGF triggers coactivation of the Ras and Cdc42, leads to activation of PI3K and NF-κB, eventually promotes TG2 expression. The overexpressed TG2 activates Src tyrosine kinase through its interaction with keratin-19, resulting in aberrant cell growth and enhanced tumorigenicity (Li et al., 2010).

#### 5.2.3 Role of TG2 in TGF-β and IL-1β pathway

Keunhee Oh et al. described the correlation between TG2 expression and IL-6 production and found that TG2-mediated IL-6 expression contributed to the tumor growth and distant liver and lung metastasis in breast cancer cells (Oh et al., 2011). In subsequent studies, they reported that IL-1β could induce IL-6 production in TG2-overexpressed cells in an NF-κB-, JNK-, and PI3K-dependent manner and described the function of IL-1β/TG2/IL-6 signaling pathway in regulating EMT, stemness and invasion (Oh et al., 2016). It's worth noting that treating cells with TGF-β could also aggravate IL-6 expression in TG2-overexpressing MCF7 cells (Oh et al., 2016).This was consistent with a previous report that demonstrated TG2 is a downstream effector of TGF-β-induced EMT in TG2-overexpressing MCF-10A cells (Kumar et al., 2010). Despite the differences of cell lines, these studies fully proved that TG2 was under the regulation of TGF-β. Moreover, Poly (ADP-ribose) polymerase 3 (PARP3) was reported to stimulate TG2 expression under the regulation of TGF-β. After activated by TGF-β-induced ROS, PARP3 promoted EMT by targeting TG2-Snail-E-cadherin axis in MCF10A cells. Considering the function of PARP3 and ATM in response to damage and the above-mentioned ATM-dependent activation of NF-κB/TG2 pathway, it is speculated that PARP3 contributed to an ATM-NF-κB-TG2-Snail axis to trigger TGF-β-induced EMT, which required further research.

## 6 Future perspectives: targeting TG2

TG2 expression is markedly upregulated in hormone receptor-positive (HR+) and human epidermal growth factor receptor-2 (Her-2+) breast cancer cells relative to their normal breast epithelial counterparts, which was a finding consistently observed across various cell lines and corroborated by clinical sample analyses (Xu et al., 2022). Therefore, targeting TG2 has emerged as a promising therapeutic strategy for breast cancer, given its multiple roles in regulating breast cancer metastasis and drug resistance. Used of various TG2 inhibitors have been reported in different diseases (Tempest et al., 2021; Song et al., 2017). Among them, ZED1227 and cysteamine have been applied in clinical trials (Schuppan et al., 2021; Verny et al., 2017). However, only several TG2 inhibitors have been verified in breast cancer (Kim et al., 2006; Bianchi et al., 2021; Wang et al., 2022; Wang and Griffin, 2013; Robitaille et al., 2008; Kim et al., 2009). The drug names, cell lines and their correspondent phenotypes in their original studies are summarized in Table1.

**TABLE 1:** Existing TG2 inhibitors in breast cancer.

Drug name	Cell lines	Phenotypes	References
CTM	MDA-MB-231and MCF/DOX	Drug resistance	Shinde et al. (2020)
R2 peptide	MDA-MB-231and MCF/DOX	Drug resistance	Shinde et al. (2020)
NC9	MCF-7	Cell movement	Chen et al. (2013)
ERW1041E	SKBR3	Cell movement	Bianchi et al. (2021)
R283	MDA-MB-231	Cell migration	Adams et al. (2020)
R294	MDA-MB-231	Cell migration	Adams et al. (2020)
Calphostin C	MDA-MB-231	Apoptosis	Ai et al. (2012)
Glucosamine	MDA-MB-231and MCF/DOX	Drug resistance	Wang et al. (2012)
AA9	MCF-7 and MDA-MB-231	Apoptosis	Alpay et al. (2015)

MCF/DOX, doxorubicin- resistant MCF7 (MCF-7/DOX); CTM, cysteamine.

Based on existing studies, we proposed three promising directions for further studies of TG2 in breast cancer (Sung et al., 2021). Despite a detailed description of TG2-mediated NF-κB signaling pathways, the mechanism of TG2-regulated metastasis and drug resistance should be studied in more depth. TG2-related issues that have been described in other cancers but not have proven comprehensively in breast cancer can provide reference. For instance, the role of TG2 in exosome, autophagy, PD-1/PD-L1 inhibitor resistance and cancer stem cells differentiation are topics worthy of further investigation (Chaffer and Weinberg, 2011). The high expression of TG2 was associated with metastasis and increased drug resistance, suggesting its potential prognostic significance. Although some researches have described its prognostic correlation preliminarily, the prognostic value of TG2 still requires in-depth investigations in breast cancer patients with different metastatic organs or resistances of different drugs (Lu and Kang, 2019). The combination of TG2 inhibitor and DOX has been well validated. However, the combined effects of TG2 with other first-line chemotherapy drugs in clinic such as taxanes, platinum and cyclophosphamide need to be further evaluated *in vitro* and *in vivo*. In addition, the combination of TG2 inhibitor and PD-1/PD-L1 inhibitor seems to be a potential solution to overcome PD-L1 inhibitor-resistance in PD-L1 (+) TNBC patients, which requires further objective appraisals. In summary, TG2 is a single target that modulates multiple pathways and functions in tumors. The development of effective TG2 inhibitors could change the way cancer is treated and therefore offers huge research potential.
